# Probiotic supplementation prevents stress-impaired spatial learning and enhances the effects of environmental enrichment

**DOI:** 10.3389/frmbi.2025.1454909

**Published:** 2025-03-05

**Authors:** Cassandra M. Flynn, Lara M. Blackburn, Qi Yuan

**Affiliations:** Biomedical Sciences, Faculty of Medicine, Memorial University of Newfoundland, St. John’s, NL, Canada

**Keywords:** gut-brain axis, inflammation, probiotic, spatial learning, stress

## Abstract

Probiotics are live microorganisms that offer health benefits, influencing the microbiota-gut-brain axis. Probiotics can improve cognitive functions, including learning and memory, by modulating the gut microbiota, reducing inflammation, and producing neuroactive substances. This study examined the effects of probiotic supplementation prior to chronic stress or enrichment (EE) treatment on cognitive function and brain physiology. Rats received probiotics or control diet starting at 6 months of age for 3 months. They were then randomly assigned to unpredictable stress, or EE for 6 weeks, with a home cage control group on a control diet included. Results showed that probiotic supplementation prevented spatial memory impairments induced by chronic stress and enhanced learning when combined with EE. These behavioral improvements were linked to increased gut microbiome diversity. Higher levels of the microglia marker Iba-1 were found in the stressed group compared to the EE group in the locus coeruleus, which probiotic reversed. Differences in blood-brain-barrier integrity were observed between the stress and EE groups, as indicated by albumin levels. Higher levels of tyrosine hydroxylase were observed in the hippocampus of the EE groups. The interaction of probiotic supplementation, chronic stress, and environmental EE offers a promising area for enhancing cognitive function and brain health.

## 1 Introduction

Chronic stress negatively impacts learning and behavior, and increases susceptibility to age-related diseases, including Alzheimer’s disease (AD), by disrupting cognitive processes, healing mechanisms, coping abilities, and overall quality of life (Polsky et al., 2022; Sotiropoulos et al., 2011; Torraville et al., 2023). Activation of neurobiological stress responses, such as the sympathetic nervous system and the hypothalamic-pituitary-adrenal (HPA) axis, contributes to higher morbidity and mortality rates, highlighting the profound impact of chronic stress on health and aging (Schneiderman et al., 2005; Shields and Slavich, 2017).

The locus coeruleus (LC)-noradrenergic system is critically involved in stress-related disorders, with its dysregulation negatively impacting health and cognition (Suárez-Pereira et al., 2022). Noradrenergic neurons project to the hypothalamus and key structures involved in learning and memory, therefore, stress-induced alterations in LC health and norepinephrine release can significantly impair brain function (Wang et al., 2017). Additionally, stress hormones impair hippocampal function via glucocorticoid receptors, affecting various types of memories (Lupien et al., 2018). Chronic stress also disrupts microglial function, potentially compromising brain homeostasis and contributing to anxiety phenotypes (Chen et al., 2024).

In contrast, environmental enrichment (EE) promotes cognitive health and resilience by providing cognitive, sensory, and motor stimulation that enhances brain plasticity and cognitive reserve (Mandolesi et al., 2017; Torraville et al., 2023). EE fosters neurobiological adaptations, such as improved learning and memory, increased neurotrophic factors, enhanced hippocampal neurogenesis, and improved synaptic connections (Mora et al., 2007; Van Praag et al., 2000; Segovia et al., 2009; Leggio et al., 2005), which collectively fortify brain health against age-related cognitive decline and neurodegenerative diseases (Rolland et al., 2008; Marx, 2005).

Emerging research highlights the interaction between gut microbiota and stress. The gut microbiota, comprising trillions of microorganisms in the gastrointestinal tract, plays a critical role in nutrient metabolism, immune modulation, and overall health (Thursby and Juge, 2017). Maintaining a balanced gut microbiome is essential for health, as disruptions, known as microbial dysbiosis, can lead to inflammation and accelerated aging (Thevaranjan et al., 2017). Stress alters gut microbiota composition and function by affecting gastrointestinal motility, increasing gut permeability, and influencing microbial growth (Ulrich-Lai and Herman, 2009; Van Wijck et al., 2012; Galley and Bailey, 2014).

Probiotics, live microorganisms conferring health benefits, have gained attention for their potential to modulate gut microbiota composition, enhance gut health, and improve immunity (Liu et al., 2021). Lactobacillus and Bifidobacterium species, extensively studied for their stress-alleviating, gastrointestinal barrier-enhancing, and anti-inflammatory effects, may also impact neurobiological pathways involved in stress resilience and cognitive function (Arseneault-Bréard et al., 2012; Moya-Pérez et al., 2017; Liu et al., 2020), though the exact mechanisms remain unclear.

Our study investigates whether probiotic supplementation can alter the brain’s response to chronic stress and amplify the positive effects of EE on cognition, and brain health. By exploring these interactions, we aim to uncover mechanisms through which probiotics may mitigate stress-induced effects and cognitive resilience and overall brain health.

## 2 Materials and methods

### 2.1 Subjects and ethics statement

Sprague-Dawley rats of both sexes were used. Rats were kept in a standard 12-hour light–dark cycle, with food and water *ad libitum* except during the probiotic feeding stage. The regular water was filtered three times (0.2 microns) and the diet (Catalog number: Teklad 2018) was irradiated. Experimental procedures were approved by the Institutional Animal Care Committee at Memorial University of Newfoundland and followed the Canadian Council’s Guidelines on Animal Care.

### 2.2 Experimental design

**Figure 1:**
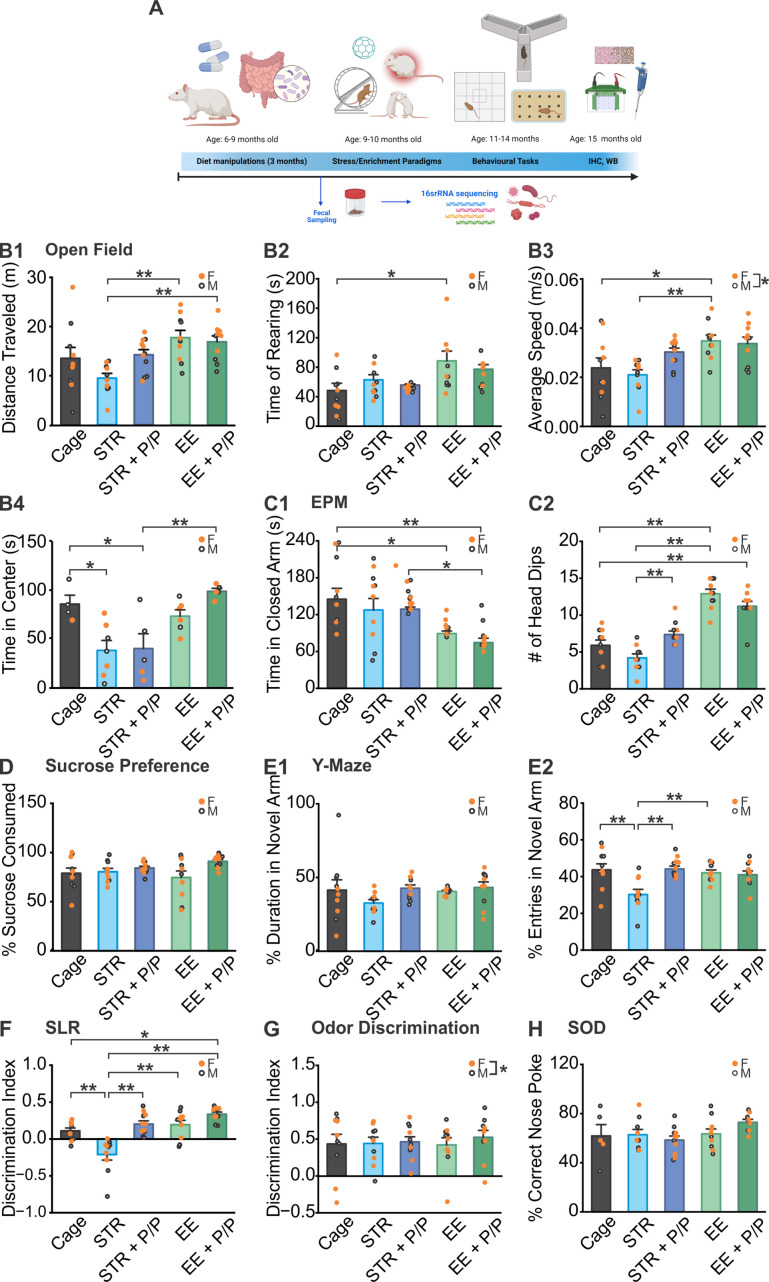
The effects of probiotics in combination with stress and EE on behavioral tasks. **(A)** Schematic of experimental design timeline. A separate cohort with probiotic or control diet was used for fecal sampling. **(B1–B4)** Distance traveled **(B1)**, time spent rearing **(B2)**, average speed **(B3)** and time spent in center **(B4)** measurements in an open field maze test. **(C1, C2)**. Time spent in the closed arm **(C1)** and number of head dips over the edge **(C2)** in an elevated plus maze (EPM) test. **(D)** Percentage of sucrose solution consumption over a 24 hr period. **(E1, E2)**. Percentage of time **(E1)** and number of entries **(E2)** in the novel arm in a Y-maze test. **(F)** Discrimination index in a spontaneous location recognition (SLR) task. **(G)** Discrimination index in an odor discrimination task. **(H)** Percentage of correct nose poke in standard object discrimination (SOD) task. STR, stress; EE, environmental enrichment; P/P, probiotic + prebiotic; N (except B4): Cage: 5F/5M; STR: 5F/5M; STR + P/P Diet: 6F/5M; EE: 5F/5M; EE + P/P Diet: 6F/4M. N **(B4)**: Cage: 1F/3M; STR: 4F/3M; STR + P/P Diet: 2F/3M; EE: 4F/2M; EE + P/P Diet: 3F/2M *p < 0.05, **p < 0.01. Source: **(A)** Created with BioRender.com.

**Figure 1A** shows the flow of the experiment. Rats underwent diet supplementation at 6 months of age, for 3 months. Following that, animals underwent randomly assigned stress and EE, or cage control paradigms daily for 6 weeks, from 9–10 months of age. Animals then underwent a battery of behavioral tasks to assess general behavior and cognitive function before being sacrificed for Immunohistochemistry and Western Blot assays. A separate cohort of animals had fecal collection following probiotic supplementation, for 16S rRNA sequencing and gut microbiome analysis. Surfaces were sterilized with 70% ethanol, cages and toys were autoclaved.

Rats were randomly assigned to five conditions, (1) Cage (control without stress or EE manipulation + control diet), (2) Stress (STR) (stress paradigm + control diet), (3) STR + P/P (stress paradigm + probiotic/prebiotic diet), (4) EE (EE paradigm + control diet), or (5) EE + P/P (EE paradigm + probiotic/prebiotic diet). Groups were sex balanced.

### 2.3 Probiotic diet supplementation

ProBiotic-4, comprised of *Bifidobacterium Lactis* (50%), *Lactobacillus casei* (25%), *Bifidobacterium bifidum* (12.5%), and *Lactobacillus acidophilus* (12.5%), were purchased from Swanson (Fargo, ND, USA). Rats received ProBiotic-4 (3 × 109 CFU) once daily for three months at 6–9 months of age, dissolved in 30 ml water (Yang et al., 2020), for a final concentration of 10.9 CFU/ml daily. The prebiotics were mixed with prebiotic oligofructose/FOS Orafti^®^ P95 powder (200 mg/kg; Quadra Chemicals) (Li et al., 2023), to improve the effectiveness of probiotics (Roy and Dhaneshwar, 2023). Regular water was provided only after the probiotic mixture was fully consumed, ensuring that the rats received the entire dose of the probiotics. Control rats received regular water only. Body weight was measured bi-weekly.

### 2.4 Stress paradigms

Stress paradigms were implemented using a chronic unpredictable stress paradigm for six weeks from 9–10 months of age following a protocol similar to those previously described (Yalcin et al., 2005; Zhou et al., 2019; Strekalova et al., 2022). Stressors were applied for the same 2 hours per day for the duration, including restraint, wetted bedding, tilted cage, and an irregular light cycle.

### 2.5 EE paradigms

EE paradigms were implemented by placing 4–5 animals together in a 60 × 60 × 50 cm Plexiglas play arena for 2 hours per day for six weeks, at 9–10 months of age (Leggio et al., 2005). The play arena contained toys, exercise equipment, and treats. Males and female animals were separated into different arenas.

### 2.6 Fecal collection

Fecal samples were collected from a separate set of female rats following probiotic or control diet feeding. Animals were placed in clean autoclaved cages, where freshly voided fecal material was collected in sterile centrifuge tubes before being stored at −80°C until DNA extraction.

### 2.7 DNA extraction from feces

Isolation of microbial genomic DNA from each animal’s stool sample was performed using the QIAamp PowerFecal Pro DNA Kit (Qiagen, Hilden, Germany) as per the manufacturer’s instruction.

Prior to storage, quality control measures were implemented to evaluate DNA purity via the Thermo ScientificTM NanoDropTM One Spectrophotometer (Thermo ScientificTM 840274100). The DNA concentration from 1μl of each sample was determined by absorbance at 260nm (A260), and the purity was estimated by determining the A260/A280 ratio with the Nanodrop spectrophotometer, then samples were stored in -20°C until shipment.

### 2.8 Microbiome analysis

16S rRNA sequencing was performed at the Integrated Microbiome Resource (Dalhousie University, Halifax, Canada). Only samples from female rats were included. The V6–V8 bacterial region of 16S rRNA genes was analyzed as previously described (Comeau et al., 2011). The library was sequenced on an Illumina MiSeq platform.

### 2.9 Behavioral testing

#### 2.9.1 Exploratory, anxiety and depressive behavior

Rats were given one 10-minute trial to explore an open field (60 × 60 × 40.5 cm^3^) and recorded with ANY-Maze software (Stoelting). Distance traveled, time spent rearing (including free and supported rearing), average speed, and time spent in center (indicating the level of anxiety) were recorded and analyzed offline as previously described (Omoluabi et al., 2021).

Anxiety was measured by the open field behavior, a 5 minute Elevated Plus Maze trial (50 × 10 cm^2^/arm with an 11 × 11 cm^2^ central platform, 38 cm walls on the closed arms), where time spent inside closed arms vs. open arms as well as head dips over the open arms were analyzed. Depressive behavior was measured by 24 hours sucrose (0.75%) percentage consumption.

#### 2.9.2 Spatial memory assessments

To assess short-term spatial memory, the Y-maze was used and to assess long-term spatial memory, animals underwent the spontaneous location recognition (SLR) task.

In the Y-maze, animals explored a black opaque Plexiglas Y-shaped maze with three arms 120 apart (50 cm × 16 cm × 32 cm^3^). For the training phase, animals had a 15-minute trial in which they were allowed to freely explore two of three arms. Which of the arms was closed for this training phase was counterbalanced between groups and animals. For the testing phase, animals were re-placed into the same “start” arm for another 15-minute trial and allowed to explore the full maze with all three arms open, with the previously closed arm considered to be the “novel” arm. Number of entries into the arms and duration in each arm was recorded for analysis (Dellu et al., 1992).

For the SLR task, rats were given 10 minutes in an open arena (60 × 60 × 40.5 cm^3^) with three identical objects (1, 2, and 3) placed at specific positions. During testing (24 hours later), rats were placed in the same arena with two identical objects, one in the same position as Object 1 (a familiar location, F), the other midway between previous Objects 2 and 3 (a novel location, N). The discrimination ratio was the difference between time spent at the novel and familiar objects over the total time spent on both objects (Bekinschtein et al., 2013).

#### 2.9.3 Odor discrimination task

Discrimination of similar odors was tested with an odor detection and discrimination task (ODAD), using perforated micro-centrifuge tube containing filter paper with 60 μl of odorant or mineral oil. The first three trials used odorless mineral oil, the next three trials used odor 1 (O1, 1-heptanol, 0.001%), and the last trial used an odor mixture that had a similar smell to O1 (O2, 1-heptanol and 1-octanol in a 1:1 ratio, 0.001%). The discrimination index was the ratio of the sniffing time difference between the O2 and the third presentation of O1 to the total sniffing time (tO2-tO1-3)/(tO2+tO1-3).

#### 2.9.4 Odor associative learning

Rats were food deprived for 3–6 days before the onset of the experiments and food deprivation continued during the experiment. Rats were placed in an open arena (60 × 60 × 40.5 cm^3^) with 2 scented sponges, and Reese’s puff cereal was used as a food reward. This procedure consisted of a habituation phase, followed by an associative training phase. In the habituation phase, rats were exposed to an unscented sponge placed in random locations, baited with food.

In the associative training phase, rats were placed in a designated home corner and presented with 2 scented sponges (locations varied each trial randomly) and given a maximum of 300 s to retrieve the cereal pellet from a retrievable center hole in one scented sponge. Percentage of correct responses was counted as the number of correct responses over the number of total nose pokes.

### 2.10 Immunohistochemistry and imaging

LC tissue was extracted after decapitation and stored in 4% paraformaldehyde before being transferred to 20% sucrose in 0.1 M phosphate-buffered saline (PBS). Fifty μm sections at 150 μm intervals were collected in PVP cryoprotectant for free-floating IHC.

All histology and IHC followed established procedures (Ghosh et al., 2019; Omoluabi et al., 2021). Primary antibodies used included: Ionized calcium-binding adaptor molecule 1 (Iba1) (019-19741, Wako, 1:2000), and Albumin (16475-l-AP, ProteinTech, 1:1000). Alexa Fluor secondary antibodies (Invitrogen, 1:1000) were used.

Bright-field and fluorescence microscopy used an Olympus BX53 (Olympus) and EVOS M5000 imaging system (Thermo Fisher Scientific), respectively. Image analysis was conducted with ImageJ. The light intensity and exposure parameters were standardized across all captured images. In the LC, the numbers of positive stained cells for Iba-1 were counted and normalized to the region of interest (/mm^2^). Albumin expression was measured as the mean density of the fluorescence in the LC, normalized to the background level in the lateral vestibular nucleus (Kelly et al., 2019). Three sections of each marker within the same rostral to caudal range were used from all animals and counts from the two hemispheres were averaged. Analysis was conducted by experimenters that were blind to the experimental conditions.

### 2.11 Western blotting

Hippocampal tissue was extracted after decapitation and stored frozen. Brain tissue processing followed established protocols (Morrison et al., 2013). Total protein concentration was quantified by standard Pierce BCA protein assay kit (Thermo Scientific, 23225). Equal amounts of protein (20 μg) were separated by SDS-PAGE on 10% gels and were then transferred to Immobilon-P Transfer PVDF membranes (Merck Millipore, IPVH00010). Following transfer, the membranes were briefly rinsed with 1× low salt TBS-T (containing 1.5M NaCl, 1M Tris Base and 0.1% Tween 20) and blocked for 1 hr with 5% nonfat skim milk at room temperature. They were then incubated for 2 hrs at room temperature with the following antibodies: Glucocorticoid receptor (GR; AB92627, Abcam, 1:2000), Tumour Necrosis Factor alpha (TNFa; AB6671, Abcam, 1:2000), tyrosine hydroxylase (TH; MAB318, Millipore Sigma, 1:2000). The membranes were rinsed in TBS-T (containing 5M NaCl, 1M Tris Base and 0.1% Tween 20) and incubated for 1.5 hrs at room temperature, with either horseradish peroxidase-labeled anti-rabbit immunoglobulin G (IgG; 31460, 1:4000) or anti-mouse IgG (31430, Thermo Fisher Scientific, 1:4000). The protein bands were visualized using chemiluminescent substrate (ThermoFisher Supersignal West PICO, 34577) on a digital image scanner (ImageQuant LAS 4000) and quantified with the ImageJ software.

### 2.12 Statistical analysis

All data are shown as mean ± standard error of the mean. Statistical analysis was conducted with OriginPro 2022b software. The 16S rRNA data were analyzed using statistical tools provided by MicrobiomeAnalyst.ca. For the analysis of the sequencing data, QIIME used was for initial quality filtering, operational taxonomic unit (OTU) picking, and taxonomic assignment. Following this, in MicrobiomeAnalyst, we applied several data processing steps to ensure the integrity and accuracy of our results. Features with identical values across all samples were excluded to enhance differential analysis, and those appearing in only one sample were removed as artifacts. We employed a low count filter, setting a minimum count threshold of 4, with a 20% prevalence filter to exclude features likely resulting from sequencing errors or low-level contamination. Additionally, we applied a low variance filter, excluding features with minimal variance across conditions, as measured by the inter-quantile range (IQR). Data were scaled using the default Total Sum Scaling (TSS) method to normalize sample sizes. Importantly, the data were not rarefied or transformed prior to analysis.

Alpha diversity was assessed using the Chao1 index, and differences between the two groups were evaluated with a t-test. Beta diversity was quantified using the Bray-Curtis index, and statistical significance was determined via PERMANOVA. Additionally, heat tree analysis, which leverages the hierarchical structure of taxonomic classifications, was used to visualize and compare taxonomic differences between microbial communities. The differences were quantitatively represented using median abundance and statistically evaluated using the non-parametric Wilcoxon Rank Sum test, with FDR (false discovery rate)-adjusted P-value cutoff set at 0.1, and log LDA (linear discriminant analysis) score set at 2.0. Behavioral results were analyzed by two-way ANOVA (group × sex). IHC and Western blotting results were analyzed by three-way ANOVA (treatment × diet × sex). *Post-hoc* Tukey tests were used for group comparisons. Homogeneity of variance was assessed with Levene’s test. Normality of the data was assessed with Shapiro-Wilk test, and met before t-tests or ANOVAs. One outlier in the TH measurement with Western blotting (> mean ± 2SD) was removed from the final analysis.

## 3 Results

### 3.1 EE increased exploratory behavior while stress induced anxiety

The open field task revealed significant differences between groups in distance traveled (*F*_4,41_ = 4.732; *p* = 0.0031; **Figure 1B1**), rearing (*F*_4,41_ = 3.781; *p* = 0.01; **Figure 1B2**), speed (*F*_4,41_ = 5.119; *p* = 0.0019; **Figure 1B3**), and time spent in center (*F*_4,17_ = 8.439; *p* = 6.09E-4; **Figure 1B4**). Both EE groups regardless of diet type traveled significantly longer distance than the STR group (*p* < 0.01). In terms of exploration, EE animals spent significantly more time rearing compared to cage control animals (*p* < 0.05). Similarly, the EE groups had higher travel speed compare to cage (*p* < 0.05), and STR (*p* < 0.01) groups. A sex difference was observed (*F*_1,41_ = 4.395; *p* = 0.042), with females moving at a higher speed than males. STR animals showed a significantly lower time spent in the center compared to cage groups (p < 0.05), regardless of diet type. There was no significant diet × sex × time interaction on body weight (**Supplementary Figure 1**).

Anxiety levels were then measured using the elevated plus maze, with a significant difference observed between groups for time spent in the closed arm (*F*_4,41_ = 5.394; *p* = 0.0014; **Figure 1C1**), no significance was observed between sexes. EE animals spent significantly less time in the closed arm than caged animals regardless of diet type (control diet, *p* < 0.05; probiotic diet, p < 0.01). When measuring head dips, another indicator for anxiety level, a significant difference was found between groups (*F*_4,41_ = 35.7789; *p* = 7.18E^−13^; **Figure 1C2**). Similarly, EE and EE + probiotic groups showed significantly higher levels of this behavior over cage (*p* < 0.01). Interestingly, probiotic supplementation significantly increased this exploratory behavior in STR animals compared to control diet-fed ones (*p* < 0.01). No sex differences were observed.

The sucrose preference test assessed the level of anhedonia, as animals with anhedonia are less interested in palatable food. Our result revealed no significant differences in the consumption of sucrose water between groups, or sexes (**Figure 1D**).

### 3.2 Probiotic supplementation rescued the spatial learning deficiency in STR rats, and enhanced memory of EE rats

In the Y-maze task, a group × sex interaction was observed in duration of time spent in the novel arm (*F*_4,41_ = 2.908, *p* = 0.033; **Figure 1E1**), although no significant difference was found in the *post-hoc* Tukey test. However, there was a significant difference between groups for the number of entries in the novel arm (*F*_4,41_ = 6.441, *p* = 4.06E^−4^; **Figure 1E2**). Following stress, animals displayed a deficit compared to cage control groups (*p* < 0.01) and EE groups (*p* < 0.05 or *p* < 0.01), and this was prevented by probiotic feeding (*p* < 0.01).

In the SLR task, there was a significant difference between groups (*F*_4,41_ = 14.508, *p* = 1.83E^−7^; **Figure 1F**). A memory deficit was observed for STR animals compared to cage control animals (*p* < 0.01). Like the Y-maze task, this deficit was restored, with a significant increase in discrimination observed in STR + probiotic group (*p* < 0.01). Probiotic feeding in EE animals led to a significantly better discrimination ability than cage (*p* < 0.05) and STR (*p* < 0.01) animals.

Olfactory impairments were tested using a similar odor discrimination task. No differences were observed between groups. Interestingly, in general, male rats performed better than female rats (*F*_1,41_ = 4.2, *p* = 0.046; **Figure 1G**). Additionally, an olfactory dependent rewards association task revealed no differences between groups or sexes (**Figure 1H**).

### 3.3 Probiotic supplementation enriches microbiome diversity in the gut

**Figure 2:**
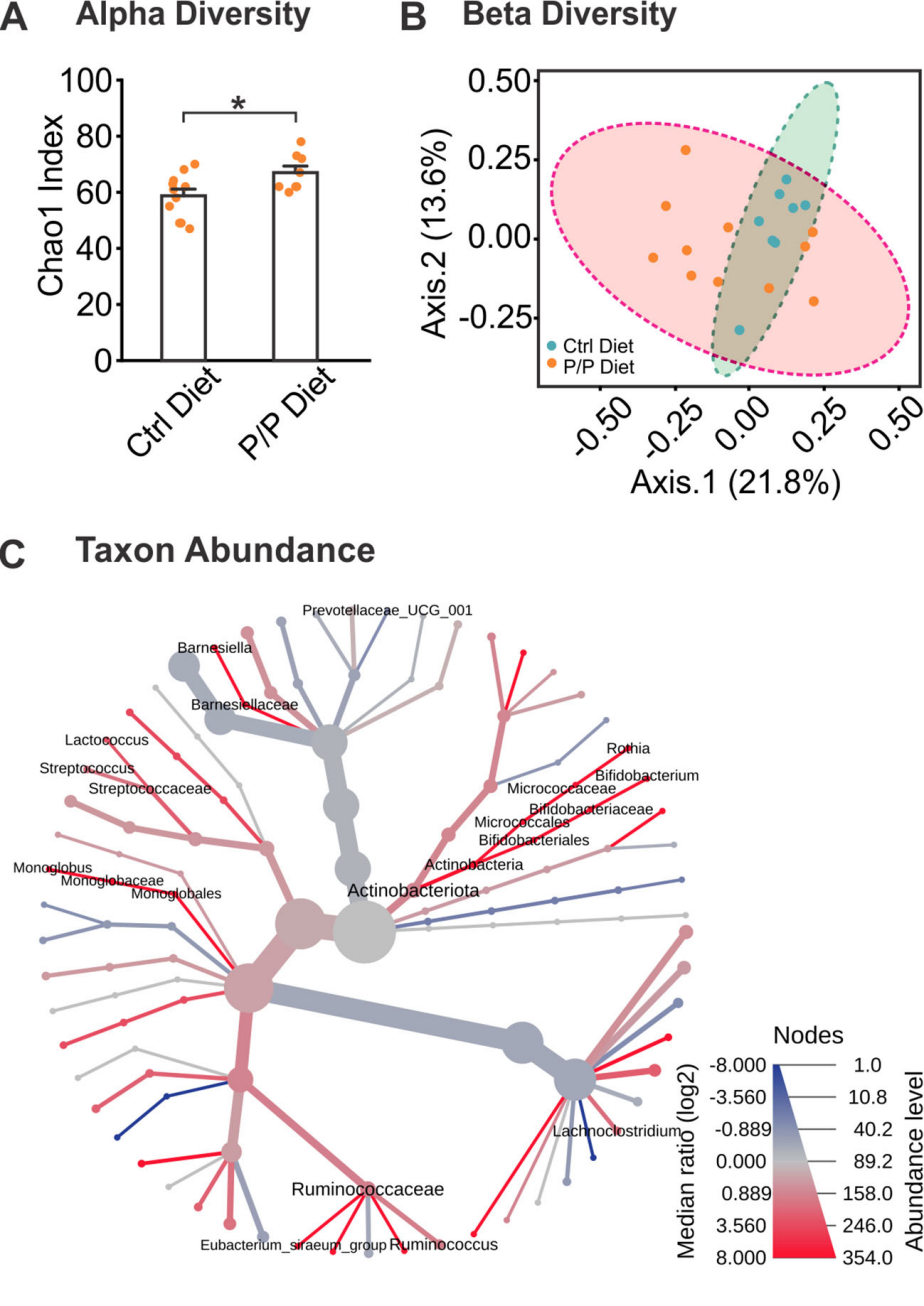
Alterations of microbiota in diversity and abundance following probiotic supplementation. **(A)** Chao1 analysis of Alpha Diversity levels between control diet and probiotic-fed groups. **(B)** Ordination plot of Beta Diversity index between diet groups, using Bray-Curtis index distance method. **(C)** A heat tree demonstrating bacterial abundance differences between diet groups. Labeled branches represent a significant abundance level between groups. N: Control diet: 11F; Probiotic diet: 8F. *p < 0.05, **p < 0.01.

Levels of alpha diversity and beta diversity were obtained from 16S rRNA sequencing. Using the Chao1 index, alpha diversity level, a metric measuring the richness (number of taxa), or evenness (relative abundance of those taxa) was analyzed. A significant increase in microbiome alpha diversity was observed in the probiotic diet groups compared to control diet groups (*t =* −2.405, *p* = 0.027; **Figure 2A**). Levels of beta diversity, representing the diversity and variability of community composition, showed a non-significant increase in beta diversity was observed in the probiotic diet groups, compared to control diet groups (*F*_17_ = 1.566, *p* =0.066; **Figure 2B**).

**Figure 3:**
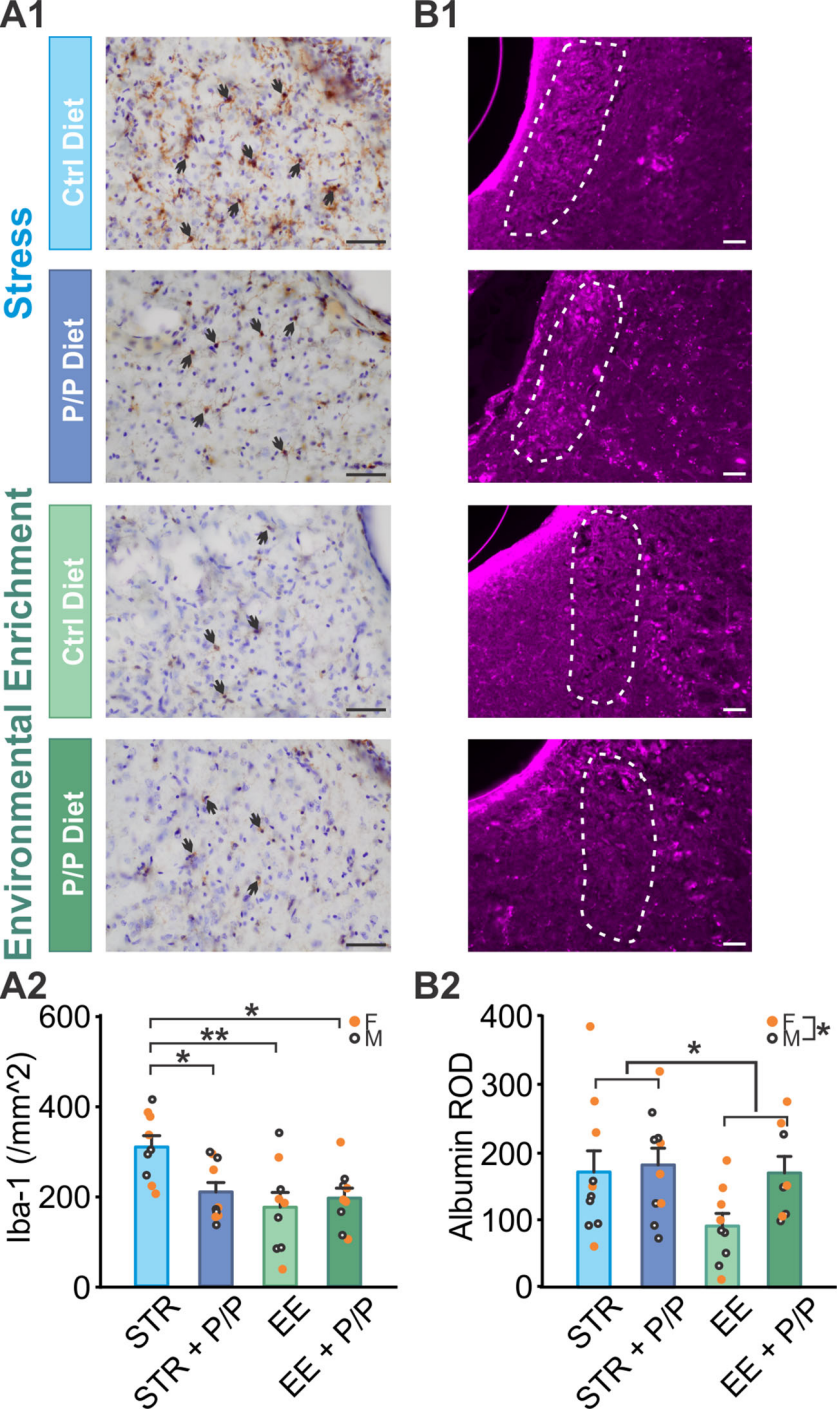
The effects of stress, EE and diet manipulation on microglia and albumin levels in the locus coeruleus (LC). **(A1)** Example images of Iba-1 staining in the LC. Arrows indicate positively stained cells. **(A2)** Number of Iba1 cells per mm^2^ in the LC. N: STR: 5F/4M; STR + P/P Diet: 5F/5M; EE: 4F/5M; EE + P/P Diet: 5F/4M. **(B1)** Example images of albumin staining in the LC. White circles indicated the LC region. **(B2)** Mean intensity of albumin staining in the LC. N: STR: 5F/5M; STR + P/P Diet: 4F/5M; EE: 5F/4M; ER + P/P Diet: 5F/4M. STR, stress; EE, environmental enrichment; P/P, probiotic + prebiotic. Scale bars: 50 µm. *p < 0.05, **p < 0.01.

Changes in the taxa species induced by Probiotic-4 mixture feeding were further assessed (**Supplementary Table 1**). At the genus level, bacterial taxa were analyzed for abundance levels between groups to observe any effect from the introduction of probiotics (**Figure 2C**). The heat map in **Figure 3C** shows average levels of each taxon and significance levels of the probiotic diet groups compared to the control diet using the Wilcoxon test. A labeled taxa is indicative of a significant difference between groups. Overall, 5 taxa genera were increased in abundance in the probiotic diet groups, and one was decreased, compared to control, demonstrating a significant difference in gut microbiome makeup from specific bacterial taxonomic groups. This includes the increase of Actinobacteria (including Bifidobacterium and Rothia species), Ruminococcaceae, Monoglobales, Streptococcaceae, and Barneseillaceae, as well as a decrease of Lachnoclostridium abundance.

### 3.4 Stress increased levels of microglia in the locus coeruleus compared to EE, which was reversed by probiotic supplementation

LC is critically involved in stress response and novelty-associated EE (Prokopiou et al., 2022; Suárez-Pereira et al., 2022). We therefore tested LC inflammation levels using Iba-1 microglial marker. We also measured blood-brain barrier (BBB) integration with albumin staining. Stress has been shown to impair BBB and probiotic supplementation has been associated with improved BBB (Torraville et al., 2023).

For Iba-1 levels, there was a significant treatment × diet interaction (*F*_1,29_ = 4.866, *p* = 0.035; **Figures 3A1, 3A2**), with STR animals showing a significant increase compared to animals in EE groups (*p* < 0.05 or *p* < 0.01) which was reserved by probiotic supplementation (*p* < 0.05). Albumin levels showed a significant difference between treatment groups (*F*_1,29_ = 4.811, *p* = 0.036; **Figures 3B1, 3B2**), with significantly higher levels in STR animals compared to EE animals (*p* < 0.05). A sex difference was also observed in albumin levels (*F*_1,29_ = 4.788, *p* = 0.037), with females showing higher levels of albumin than males.

**Figure 4:**
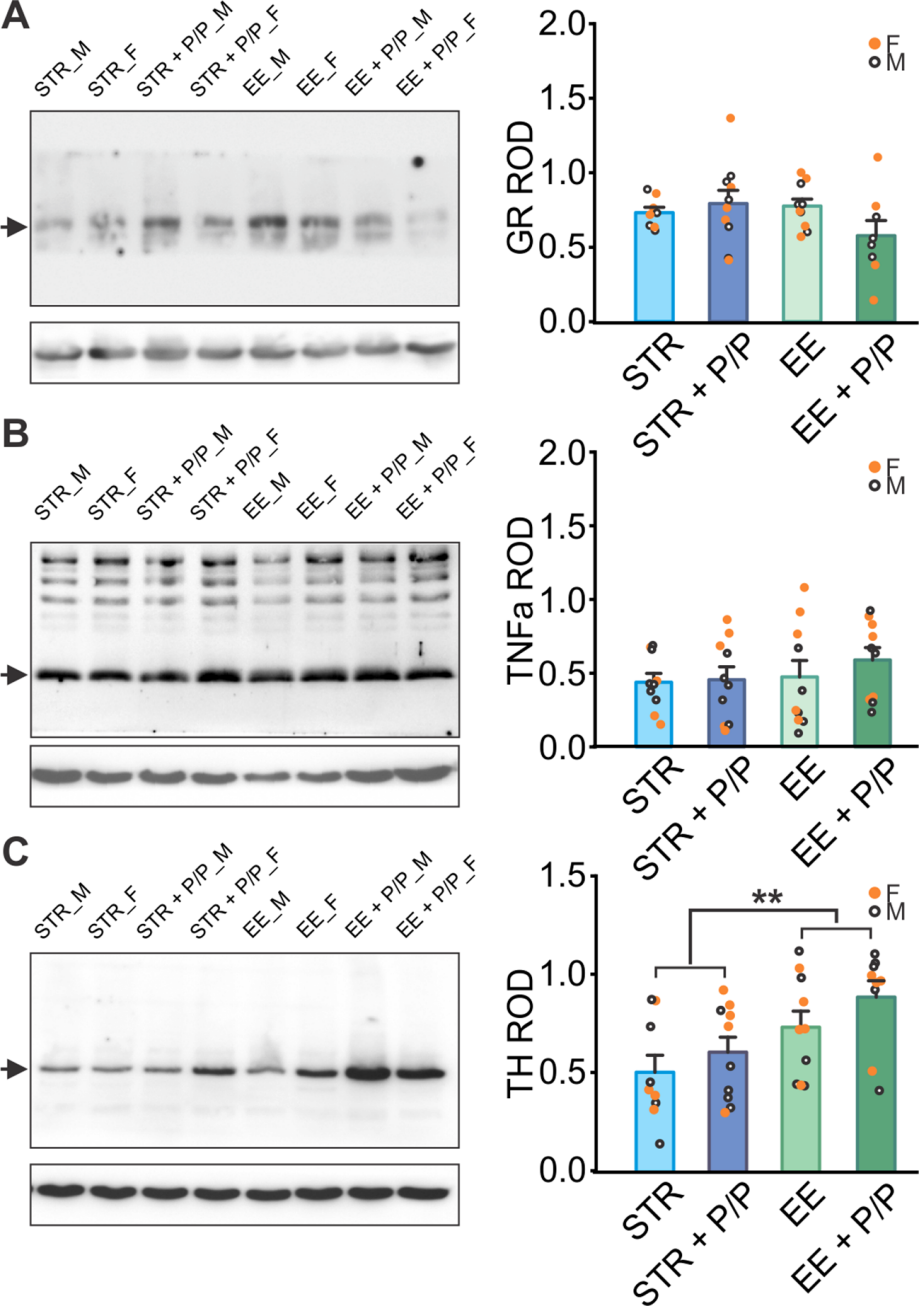
The effects of stress, EE and diet manipulation on hippocampal markers. **(A)** Representative blot and quantity of glucocorticoid receptor (GR) normalized to beta-actin levels in the hippocampus. N: STR: 4F/4M; STR + P/P Diet: 5F/5M; EE: 5F/5M; ER + P/P Diet: 4F/4M. **(B)** Representative blot and quantity of Tumour Necrosis Factor aloha (TNFa) normalized to beta-actin levels in the hippocampus. N: STR: 5F/5M; STR + P/P Diet: 5F/5M; EE: 5F/5M; ER + P/P Diet: 5F/5M. **(C)** Representative blot and quantity of tyrosine hydroxylase (TH) normalized to beta-actin levels in the hippocampus. N: STR: 5F/4M; STR + P/P Diet: 5F/5M; EE: 5F/5M; ER + P/P Diet: 5F/4M. STR, stress; EE, environmental enrichment; P/P, probiotic + prebiotic. **p < 0.01.

Furthermore, measurements of GR (**Figure 4A**), inflammation marker TNFa (**Figure 4B**), and norepinephrine marker TH (**Figure 4C**) were conducted in the hippocampus. There were no significant differences among groups in the levels of GR and TNFa. TH levels were higher in the EE treated rats than the STR rats (*F*_1,30_ = 8.941, *p* = 0.006; **Figure 4C**).

## 4 Discussion

Current research underscores the intricate relationship between stress and the gut microbiota, with probiotic interventions emerging as a promising strategy to modulate these interactions. Our study explored the effects of administrating probiotics prior to stress or EE on cognition and inflammation. We observed increased anxiety in animals exposed to chronic stress, while the EE paradigm promoted exploration and reduced anxiety. Probiotic supplementation enhanced gut microbiome diversity, alleviated anxiety, prevented spatial learning impairment in stressed rats, and boosted learning in the EE group. Additionally, chronic stress increased microglial activity (Iba-1) in the LC, a change that probiotics prevented. BBB integrity was lower in stressed animals compared to those in the EE group. Higher tyrosine hydroxylase levels in the hippocampus of enriched groups may correlate with better LC function and axonal release. These findings suggest a mechanistic link between reduced stress, improved gut health, and decreased brain inflammation. Chronic stress can significantly impact the immune system through the microbiota-gut-brain axis, as evidenced by systemic inflammatory increases coinciding with stress and changes in microbiota composition and barrier function (Pasiakos et al., 2016; De Palma et al., 2015; Moya-Pérez et al., 2017). In addition, this response triggers a dysregulated HPA axis, as shown by elevated corticosterone and adrenocorticotropic hormone in germ-free mouse studies (Ackerman et al., 1978). Stress-induced inflammation can exacerbate psychiatric disorders like depression and anxiety, further disrupting gut microbiota composition and creating a vicious cycle of stress and dysbiosis (Li et al., 2019). This cycle is associated with increased intestinal permeability, often referred to as “leaky gut,” and increased BBB permeability, which we observed in our study. This barrier weakening can lead to systemic inflammation and contribute to various health issues (Chatzaki et al., 2003; Dodiya et al., 2020; Welcome and Mastorakis, 2020).

Probiotics, beneficial live microorganisms, can positively influence the gut microbiota and, by extension, the gut-brain axis (Arseneault-Bréard et al., 2012; Moya-Pérez et al., 2017). They help restore the balance of gut microbiota disrupted by stress by outcompeting pathogenic bacteria and promoting the growth of beneficial microbes, thereby improving overall microbiota composition (Dimidi et al., 2017). This restoration can reduce inflammation and enhance both gut and mental health (Arseneault-Bréard et al., 2012; O’Sullivan et al., 2011; Bravo et al., 2011). Notably, our study demonstrates that prior probiotic supplementation can increase animals’ resistance to chronic stress. Probiotics have also been shown to boost the production of anti-inflammatory cytokines while reducing pro-inflammatory cytokines, thereby modulating immune responses and reducing chronic inflammation (Virk et al., 2024). Additionally, probiotics can increase the expression of tight junction proteins, reducing intestinal permeability and preventing systemic inflammation (Gou et al., 2022). Certain probiotic strains produce neurotransmitters like serotonin and GABA, which can improve mood and reduce anxiety (Duranti et al., 2020; Akram et al., 2024). Importantly, probiotic feeding has been associated with increased levels of short-chain fatty acid (SCFA)-producing bacteria, which can further promote anti-inflammatory cytokine release and enhance tight junction integrity (Markowiak-Kopeć and Śliżewska, 2020). In our study, levels of beneficial Bifidobacterium species were found to be increased through probiotic feeding. Levels of SCFA-producing genera, such as Ruminococcaceae (Grabinger et al., 2019), were found to be increased following feeding, while levels of Lachnoclostridium could influence immune system responses (Yu et al., 2024).

Dietary interventions involving probiotics have shown promise in clinical settings, particularly as adjunctive treatments for managing chronic stress, improving mental health, and enhancing overall immune function (Den et al., 2020; Mazziotta et al., 2023). This approach is especially beneficial for conditions characterized by both psychological and gastrointestinal symptoms. In our study, animals were fed probiotics prior to significant environmental manipulation. Prolonged probiotic supplementation prevented stress-induced learning deficiencies and neuroinflammation, suggesting that probiotic supplementation could serve as a preventive strategy against stress-induced physiological and psychological disorders.

In summary, the relationship between the gut microbiome and stress can be modulated through immune responses and reduced inflammation via probiotic supplementation. Incorporating probiotics into the diet is a viable strategy to mitigate the adverse effects of chronic stress on the immune system, thereby supporting better mental and physical health. One limitation of our study is the small sample size for sex-dependent analysis. Additionally, further research is needed to characterize the gut microbiome in male animals. Another limitation of our study is the lack of separate control groups for probiotics or prebiotics alone. We opted to include a single group receiving both probiotics and prebiotics, as previous research has shown that the combination, referred to as ‘synbiotics,’ provides optimal effects (Chunchai et al., 2018; Morshedi et al., 2020). In contrast, prebiotics alone have yielded mixed results, with some studies showing no effect (Kazemi et al., 2019; Alli et al., 2022) while others reported some benefits (Alli et al., 2022). Despite these limitations, our study underscores the importance of the gut-brain-immune axis in developing therapeutic interventions, offering a holistic approach to health management.

## Data Availability

The original contributions presented in the study are publicly available. This data can be found here: 10.6084/m9.figshare.28501892.
