# Supplementary material for: Probiotic supplementation prevents stress-impaired spatial learning and enhances the effects of environmental enrichment
Source: Front Microbiomes. 2025 Mar 5;4:1454909. doi: 10.3389/frmbi.2025.1454909 (PMC12993499; doi:10.3389/frmbi.2025.1454909)
Supplement: Supplementary file 1 [file DataSheet1.pdf]

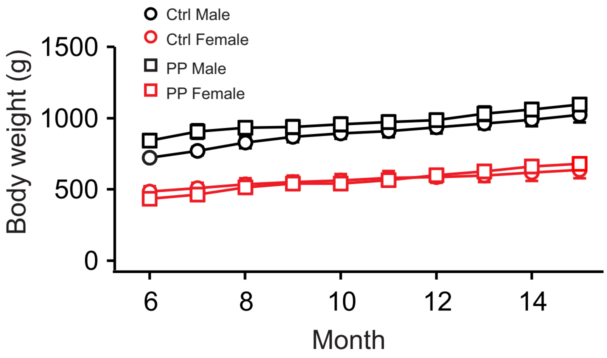

Supplementary Figure 1. Body weight measurements of control (Ctrl) and probiotic/prebiotic (PP) groups of both sexes.
